# Tailored Beta-catenin mutational approach in extra-abdominal sporadic desmoid tumor patients without therapeutic intervention

**DOI:** 10.1186/s12885-016-2704-4

**Published:** 2016-08-26

**Authors:** Danique L.M. van Broekhoven, Dirk J. Grünhagenl, Thijs van Dalen, Frits van Coevorden, Han J. Bonenkamp, Lukas B. Been, Marc H.A. Bemelmans, Sander D.S. Dijkstra, Chiara Colombo, Alessandro Gronchi, Cornelis Verhoef

**Affiliations:** 1 Erasmus MC Cancer Institute, Rotterdam, The Netherlands; 2 University Medical Center Utrecht, Utrecht, The Netherlands; 3 Netherlands Cancer Institute, Amsterdam, The Netherlands; 4 Radboud University Medical Center, Nijmegen, The Netherlands; 5 University Medical Center Groningen, Groningen, The Netherlands; 6 Maastricht University Medical Center, Maastricht, The Netherlands; 7 Leiden University Medical Center, Leiden, The Netherlands; 8 Fondazione IRCCS Istituto Nazionale dei Tumori, Milan, Italy

**Keywords:** Aggressive fibromatosis, Desmoid, Desmoid-type fibromatosis, Watchful waiting, Wait-and-see, Growth, Progression

## Abstract

**Background:**

The efficacy of the classical treatment modalities surgery and radiotherapy in the treatment of aggressive fibromatosis is presently disputed and there is a shift towards a more conservative approach. The aim of the present study is to objectify tumor growth in patients with extra-abdominal or abdominal wall aggressive fibromatosis, while adhering to a “watchful waiting” policy. Other objectives are to investigate quality of life and to identify factors associated with tumor growth, in particular the relation with the presence of a CTNNB1-gene mutation in the tumor.

**Design and methods:**

GRAFITI is a nationwide, multicenter, prospective registration trial. All patients with extra-abdominal or abdominal wall aggressive fibromatosis are eligible for inclusion in the study. Main exclusion criteria are: history of familiar adenomatous polyposis, severe pain, functional impairment, life/limb threating situations in case of progressive disease. Patients included in the study will be treated with a watchful waiting policy during a period of 5 years. Imaging studies with ultrasound and magnetic resonance imaging scan will be performed during follow-up to monitor possible growth: the first years every 3 months, the second year twice and the yearly. In addition patients will be asked to complete a quality of life questionnaire on specific follow-up moments. The primary endpoint is the rate of progression per year, defined by the Response Evaluation Criteria In Solid Tumors (RECIST). Secondary endpoints are quality of life and the rate of influence on tumor progression for several factors, such as CTNNB1-mutations, age and localization.

**Discussion:**

This study will provide insight in tumor behavior, the effect on quality of life and clinicopathological factors predictive of tumor progression.

**Trial registration:**

The GRAFITI trial is registered in the Netherlands National Trial Register (NTR), number 4714.

## Background

### Biological behavior

Desmoid-type fibromatoses are rare, non-metastasizing, locally aggressive soft tissue tumors. Aggressive fibromatoses can be located in every part of the body and are classified as extra-abdominal, abdominal wall or intra-abdominal [1, 2]. The abdominal wall is a predilection site in women of reproductive age [3]. Sporadic onset of the tumor is common, but an association with familiar adenomatous polyposis (FAP) has been documented, in particular in intra-abdominally localized aggressive fibromatoses [4]. The course of the disease is unpredictable and varies between relatively indolent, i.e. stabilization of the tumor, and progressive growth, which may halt spontaneously [5]. The reported frequency of recurrence following local treatment ranges from 5 to 63 % [6].

Genetic markers in tumor tissue have been analyzed, in particular the CTNNB1-gene. CTNNB1-gene encodes beta-catenin, a proto-oncogene involved in cell adhesion and cell transcription. Beta-catenin is a key factor in the Wnt-APC-beta-catenin pathway. On a cellular level the beta-catenin protein level is elevated in these tumors, implicating beta-catenin stabilization as a key factor in the pathogenesis of aggressive fibromatosis [7, 8]. Nuclear overexpression of beta-catenin is a histological condition used in a diagnostic. The diagnostic value is sensitive, but not specific [8–10]. Research on the CTNNB1-gene revealed 3 specific mutations, namely T41A, S45F and 45P [8, 10]. While it is yet unclear how these mutations precisely affect the aforementioned pathway in these tumors, a role in biologic behavior seems natural according to their role in pathogenesis. Several groups have analyzed CTNNB1-mutation and these mutations appear to have a prognostic value in determining the risk of recurrence in retrospective series of surgically treated patients [11–15]. Although Mullen et al. did not find a statistical significant prognostic [15], several other groups reported a higher risk of recurrence for patients with an S45F-mutation [11–13], even in multivariate analysis [12]. In addition, (surgical) trauma and hormones presumably play a role in the genesis of this tumor, as aggressive fibromatosis is known to arise in scars and in fertile females [16].

### Treatment

Treatment of aggressive fibromatosis classically involves surgery, combined with radiotherapy on indication. Literature on the effects of surgery and radiotherapy on the rate of recurrence is conflicting [17–19]. While these effects are still being questioned, treatment policies have recently turned towards a more conservative approach. Nowadays, a watchful waiting approach is being advocated by various authors and is currently the standard in European care [20–25]. Retrospective studies showed that progression usually occurs within 2 years of diagnosis. Fiore et al. [22] reported a median time till progression of 14 months, with 89 % of progression observed within 2 years, while Salas et al. [18] described a median time till progression of 20 months. In addition, these studies have also reported spontaneous regression in up to 18.5 % of the patients [18, 22].

The ability to predict tumor behavior would enable tailoring individual patient treatment. Little is known about tumor growth. Available literature is dated and descriptive, without objective measurements [16].

### Study aim

The GRAFITI study will evaluate a watchful waiting approach as an initial treatment for patients with extra-abdominal or abdominal wall aggressive fibromatosis. The primary objective is to assess tumor progression using the Response Evaluation Criteria In Solid Tumors (RECIST) [26]. We will attempt to identify patient-and tumor characteristics related to growth. A twin study is ongoing in Milan, Italy (NCT02547831). The present study proposal was designed in collaboration with the Italian study group, to facilitate a possible future merger of data.

## Design and methods

### Study design

GRAFITI was designed in collaboration with experts in sarcoma care throughout the Netherlands as a nationwide prospective observational study. All patients with extra-abdominal or abdominal wall aggressive fibromatosis are eligible for participation. Inclusion and exclusion criteria are discussed below. If not included, treatment options will be discussed by the local multidisciplinary teams. Treatment modalities include systemic treatment, surgery and radiotherapy, and individualized treatment will be chosen based on patient characteristics, tumor localization and predicted outcome.

**Fig. 1:**
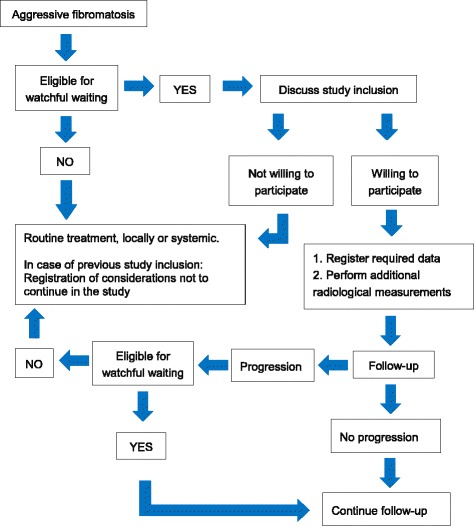
Flowchart

Patients will be treated by a watchful waiting policy and asked to complete quality of life questionnaires. During follow-up, imaging studies will be performed to monitor tumor growth. In case of growth, all treatment options will be evaluated, including continuation of watchful waiting. A switch in treatment strategy will be monitored and reasons for this switch documented (see Fig. 1).

### Primary objective

The primary objective is to assess tumor progression in terms of objectifying and monitoring growth during watchful waiting policy as an initial treatment. Ultrasound and MRI imaging will be used to determine tumor size. Tumor behavior will be scored using RECIST. Primary endpoint is the rate of progression per year, which will be measured after 5 years of follow-up.

### Secondary objectives

The secondary objective is to investigate the effect of treatment on the quality of life. During the study period, patients will be asked to complete the EORTC QLQ-C30 questionnaire five times: at inclusion and after 6,12,24 and 60 months. After a switch to active treatment, patients will remain on-study for the questionnaires. The scores will be evaluated and related to treatment policy.

Other objectives are to analyze the value of clinicopathological factors, including CTNNB1-gene mutation, in predicting progression. The reasons and considerations for active treatment will be analyzed in relation to the applicability of a watchful waiting policy.

### Study population

The study will take place in the Netherlands. All patients with extra-abdominal or abdominal wall aggressive fibromatosis are eligible for inclusion in the study. Primary and recurrent disease will be included, stratification will be done for analyses.

### Inclusion criteria

Histological evidence of aggressive fibromatosis. Capable to undergo MRI-scans and ultrasounds. Capable to understand and sign informed consent.

### Exclusion criteria

Age <18 years. Personal or family history of FAP. Intra-abdominal tumor localization. Previous treatment for the current manifestation (recurrent lesions without previous treatment are included). Severe pain or functional impairment due to the tumor (as indicated by the patient. The use of painkillers is not an exclusion criterion). Tumor progression leading to mutilation or life/limb-threatening situations, as assessed by the attending physician.

### Sample size

Based on the incidence of sporadic aggressive fibromatosis and tumor localization, we expect to include 20 patients annually, we aim to include 100 patients in 5 years. Loss to follow-up or death is not to be expected. Under the most adverse conditions, a progression rate of 50 % would result in a 95 % confidence interval (95 % CI) of 40–60 %. A progression rate of 25 % would result in a 95 % CI of 18–34 %. We consider the presented 95 % CI to be acceptable for the study.

## Methods

Participation in the study implies that the work-up does not deviate from present common practice. A contrast enhanced MRI-scan (T1 and T2 weighted) is used to determine the precise localization, size and involved structures. Subsequently, and also in line with national guidelines, the patient will undergo an ultrasound-guided, histological needle-biopsy of the soft tissue tumor, with a 14 G needle. Preferably 3 biopsies will be obtained. During the ultrasound, tumor size will be measured in three dimensions. In addition, as part of this study a quality of life questionnaire is completed by the patient.

The follow-up schedule is set for 9 outpatient-clinic visits (see Table 1). During each visit imaging studies will be performed to monitor possible growth. In addition, patients will be asked to complete a questionnaire during 5 follow-up visits. The radiology report of the ultrasound or MRI-scan will specify the maximum diameter in all 3 dimensions and the growth in relation to previous radiological examinations. When ultrasonography suggests tumor progression, an MRI-scan is additionally made as standard care and considered as the golden standard for detecting changes within the tumor.

**Table 1:** Follow-up schedule

Assessment	Enrollment	Year 1				Year 2		Year 3–5		
Month		3	6	9	12	18	24	36	48	60
History and Physical examination	x	x	x	x	x	x	x	x	x	x
MRI-scan	x		x		x		x	x		
Ultrasound	x	x		x		x			x	x
QoL questionnaire	x		x		x		x			x

In case of tumor progression, the patient will be re-evaluated. If the patient is still eligible, watchful waiting policy will be continued. If not, local or systemic treatment will be started and considerations to switch treatment strategies will be documented.

After inclusion of all patients, pathology specimens will be collected by one pathology laboratory and CTNNB1-gene analysis will be performed for all patients. If CTNNB1-mutation status is already known, this procedure will not be repeated.

### Statistical considerations

Statistical analysis will be carried out using IBM SPSS Statistics 21. Radiological measurements will be registered as a continuous variable at ratio. The average progression rate per year will be analyzed using data of all patients. The progression rate per year, defined as increase in size per tumor, using RECIST criteria, with the associated range and confidence interval, will be registered as the primary outcome. The QLQ-C30 questionnaire results in a score to classify the quality of life. This score will be registered as discrete data at ratio scale. If a score cannot be rewarded, the data of the questionnaire will be regarded as missing data. If a score is missing, but later registered scores are available, the later scores will be used in assessment of the quality of life. The overall quality of life will be calculated using data of all patients at the end of follow-up. The median value will be extracted with the associated range.

The possible influence of patient and tumor related factors on the progression rate and the quality of life are analyzed using the Kaplan-Meier method and univariable Cox-regression. Associations between variables will be explored by Chi-square analysis. Multivariate analysis will be performed if possible by means of Cox-regression. Those factors which prove to have statistical significance in univariate analyses, will be included in the multivariate analysis. The considerations for treatment will be categorized and analysis will show the occurrence of specific considerations.

The interim analysis of both primary and secondary parameters will be done after one year of follow-up on 20 patients. The analyses will be the same as described above and will be performed by the principal investigator. For all analyses, two-sided *P <* 0.050 is considered statistically significant.

## Discussion

During the last decade, there has been a shift in treatment strategy for aggressive fibromatosis from aggressive to conservative modalities. A watchful waiting policy is currently advised for extra-abdominal and abdominal wall aggressive fibromatosis [25]. Research validating the efficacy and applicability of a watchful waiting policy is limited. Mitchell et al. were the first to describe a stable phase for aggressive fibromatosis [5]. In a retrospective study of 17 patients under medical observation, all experienced at least one period of stable disease for over 6 months. A larger study by Fiore et al. evaluated 142 patients with primary and recurrent aggressive fibromatosis, treated with initial conservative treatment retrospectively [22]. Approximately 50 % of the patients did not have tumor progression after 1 year. Spontaneous regression has been reported by Salas et al. [18]. In a retrospective study analyzing 426 patients with aggressive fibromatosis, 27 patients were treated with a watchful waiting policy. Five of these patients had spontaneous remission, 16 patients stable disease and 6 patients had progressive disease. The median time to progression was 19.7 months. A recent study by Colombo et al. reported 216 patients with primary extra-abdominal (*n =* 188) and intra-abdominal (*n =* 28) disease undergoing a diversity of treatments [24]. Initial wait-and-see policy was applied in 70 patients (60 extra-abdominal) and continued till the end of follow-up in 60 %. Progression occurred in 16 of the 70 patients, mostly treated with systemic modalities. These results demonstrate the potential safety of a watchful waiting policy.

Current knowledge on predictive factors is mostly based on surgical cohorts. Age, tumor localization and tumor size have been reported as predictive factors for the risk of recurrence following surgery. A nomogram was proposed by Crago et al. [27] using all these factors in a postoperative setting.

In addition, CTNNB1-mutations are found to be a predictive factor for the risk of recurrence following surgery [12–14, 16]. The value of these factors in a postoperative setting cannot be extrapolated to a watchful waiting setting. The present study was designed to evaluate the role of these factors in relation to the progression rate in a watchful waiting setting. This information would help in determining which patients can safely undergo a watchful waiting policy, and which patients would benefit most from active treatment. The ability to predict tumor behavior would enable tailoring individual patient treatment and prevent over-or undertreatment.

The low incidence of aggressive fibromatosis presents a challenge for quality research. Collaborations between specialized institutions is essential. The prospective evaluation of predictive factors in a watchful waiting setting has been initiated by two other research groups. In France, Bonvalot et al. are conducting a similar study (ClinicalTrials.gov identifier NCT01801176). They have finished the inclusion process and are now conducting the final follow-up. In Italy, a similar study is coordinated by Colombo et al. (ClinicalTrials.gov identifier NCT02547831). This study is still open and we encourage inclusion. The present study was designed to resemble the French and Italian study, to facilitate a possible merging of the data if the inclusion rate in the studies would be disappointing. Main inclusion and exclusion criteria match for all three studies, though our study also includes patients presenting with recurrent disease.

The occurrence of aggressive fibromatosis has been related to hormonal influences and pregnancy by Häyry and Reitamo et al. [16, 28]. Although hormonal levels and receptors on the tumor have not been investigated, the occurrence of disease among fertile females is very suggestive. A recent study by van Broekhoven et al. evaluated time trends in the Dutch population [29]. Their analysis between incidence and hormonal influences did not show a positive correlation. In an attempt to evaluate the hormonal influence, data on the use of hormonal medication and history of pregnancy will be collected during the present study.

Intra-abdominal tumor depositions and personal or family history of FAP are among the exclusion criteria for the presented study. Intra-abdominal desmoid tumors are associated with FAP [30]. This association is suggestive of a different tumor biology compared to sporadic disease. In addition, intra-abdominal disease is related to a high mortality among FAP-patients and as such treated differently. To limit the risks associated with the present study, these patients are excluded from participation.

The occurrence of progression does not necessitate a switch to active treatment. In case the safety of the patient is compromised, for example due to organ involvement or increased pressure, a switch to active treatment will be recommended. In order to minimize the risk of compromised abilities due to tumor growth, the follow-up schedule allows for timely detection of tumor progression and patients with vital structures at risk will not be included in the study. The exclusion criteria prevent life threaten of functional impairment in case of tumor growth. Severe pain is considered to require continuous pain medication. Active treatment does not guarantee pain relief. As such, a watchful waiting policy should be considered and discussed in patients experiencing degrees of pain. During the study period, we will monitor the considerations in switching treatment strategies.

An interim analysis will be performed after 1 year follow-up from the first 20 patients. This analysis is designed to validate the safety of the study. If too many patients deviate from the watchful waiting policy, this policy should be questioned. Due to the benign nature of this disease, we consider it safe if over 50 % of the patients is still undergoing watchful waiting after 1 year of follow-up.

This study will provide insight in tumor behavior and clinicopathological factors predictive of tumor progression. The ability to predict tumor behavior would enable tailoring individual patient treatment.

## Abbreviations

FAP: Familial adenomatous polyposis
METC: Medical research ethics committee in Dutch: Medisch Ethische Toetsing Commissie (METC)
MRI: Magnetic resonance imaging
QoL: Quality of life
RECIST: Response evaluation criteria in solid tumors
